# Inclusion of the benefits of enhanced cross-protection against cervical cancer and prevention of genital warts in the cost-effectiveness analysis of human papillomavirus vaccination in the Netherlands

**DOI:** 10.1186/1471-2334-13-75

**Published:** 2013-02-07

**Authors:** Tjalke A Westra, Irina Stirbu-Wagner, Sara Dorsman, Eric D Tutuhatunewa, Edwin L de Vrij, Hans W Nijman, Toos Daemen, Jan C Wilschut, Maarten J Postma

**Affiliations:** 1 Department of Medical Microbiology, Molecular Virology Section, University Medical Center Groningen, University of Groningen, Antonius Deusinglaan 1, 9713 AV, Groningen, The Netherlands; 2 NIVEL Netherlands Institute of Health Service Research, Utrecht, The Netherlands; 3 Gynecologist-oncologist, Department of Gynaecology, University Medical Center Groningen, University of Groningen, Groningen, The Netherlands; 4 Health Economist, Unit of PharmacoEpidemiology & PharmacoEconomics (PE2), Department of Pharmacy, University of Groningen, Groningen, The Netherlands and Department of Epidemiology, University Medical Center Groningen, University of Groningen, Groningen, The Netherlands

**Keywords:** Cervical cancer, HPV-vaccination, Cost-effectiveness, Genital warts, Cross-protection, Pricing of vaccines

## Abstract

**Background:**

Infection with HPV 16 and 18, the major causative agents of cervical cancer, can be prevented through vaccination with a bivalent or quadrivalent vaccine. Both vaccines provide cross-protection against HPV-types not included in the vaccines. In particular, the bivalent vaccine provides additional protection against HPV 31, 33, and 45 and the quadrivalent vaccine against HPV31. The quadrivalent vaccine additionally protects against low-risk HPV type 6 and 11, responsible for most cases of genital warts. In this study, we made an analytical comparison of the two vaccines in terms of cost-effectiveness including the additional benefits of cross-protection and protection against genital warts in comparison with a screening-only strategy.

**Methods:**

We used a Markov model, simulating the progression from HPV infection to cervical cancer or genital warts. The model was used to estimate the difference in future costs and health effects of both HPV-vaccines separately.

**Results:**

In a cohort of 100,000 women, use of the bivalent or quadrivalent vaccine (both at 50% vaccination coverage) reduces the cervical cancer incidence by 221 and 207 cases, corresponding to ICERs of €17,600/QALY and €18,900/QALY, respectively. It was estimated that the quadrivalent vaccine additionally prevents 4390 cases of genital warts, reducing the ICER to €16,300/QALY. Assuming a comparable willingness to pay for cancer and genital warts prevention, the difference in ICERs could justify a slightly higher price (~7% per dose) in favor of the quadrivalent vaccine.

**Conclusions:**

Clearly, HPV vaccination has been implemented for the prevention of cervical cancer. From this perspective, use of the bivalent HPV vaccine appears to be most effective and cost-effective. Including the benefits of prevention against genital warts, the ICER of the quadrivalent HPV vaccine was found to be slightly more favourable. However, current decision-making on the introduction of HPV is driven by the primary cervical cancer outcome. New vaccine tenders could consider the benefits of cross-protection and the benefits of genital warts, which requires more balanced decision-making.

## Background

Cervical cancer is one of the most common cancers worldwide. It is caused by persistent infection with high-risk Human Papillomavirus (HPV) [1]. In Europe, two prophylactic HPV vaccines are available, a bivalent and a quadrivalent vaccine. The bivalent ASO4-adjuvanted vaccine (“Cervarix”; GlaxoSmithKline) provides protection against infection with HPV16 and 18. The quadrivalent amorphous aluminium hydroxyphosphate sulphate adjuvanted vaccine (“Gardasil” or “Silgard”; sanofi pasteur/Merck Sharp & Dohme), also protects against HPV 6 and 11, in addition to HPV16 and 18. HPV16 and 18 are the two major oncogenic HPV types and are responsible for approximately 70% of cervical cancer cases worldwide [2]. HPV6 and 11 are low-risk HPV types; these viruses are responsible for most of the cases of genital warts [3]. Obviously, the choice of which vaccine to use will depend on various factors, including the efficacy, safety and cost-effectiveness of the vaccines.

In the Netherlands, the decision of implementing HPV vaccination in the context of the National Immunization Program (NIP) has been based on the prevention of cervical cancer followed by a vaccine tender offered by the two pharmaceutical companies. Although, in the context of the Dutch drug reimbursement system, the cost of the vaccination – for both vaccines – is listed at €105 per dose, substantial price reductions are likely to be granted by the vaccine manufacturers. Therefore the predominant focus is on the actual price of the vaccines, and relevant clinical differences between both vaccines are not taken into account. These differences relate to the vaccines’ cross-protective efficacies against other, non-vaccine, high-risk HPV types and the protection against genital warts [4-6]. We argue that the decision of which vaccine to use within a country-specific immunization program should be made by taking these differences explicitly into account. In particular, the willingness-to-pay for cancer and genital warts might differ and can as such influence the acceptable cost-effectiveness ratio.

In the Netherlands, approximately 600 new cases of cervical cancer are diagnosed annually. Furthermore, the incidence of genital warts is estimated at approximately 85.8 and 121.6 per 100,000 among men and women, respectively [7]. Previously, several studies have estimated that the implementation of HPV vaccination for Dutch teenage girls, at 12 years of age, is a cost-effective intervention at around €20,000 per quality-adjusted life year (QALY) gained [8-12]. This was estimated irrespective of which specific vaccine was used [13]. The present paper aims to determine the costs and health outcomes of HPV vaccination of 12-year-old girls for both vaccines individually, considering not only the benefits of preventing cervical cancer but also those of preventing genital warts, in the context of the current cervical cancer screening program. In particular, the clinical benefits and the incremental cost-effectiveness ratios (ICERs) for both vaccines were estimated. Also, the price difference between both vaccines was estimated to achieve equal ICERs. Note that several governments, including the Dutch and UK governments,, initially did not consider the potential benefits of protection against genital warts in their decision which vaccine to choose and thus the HPV vaccine was introduced only on the basis of the prevention of cervical cancer.

## Methods

### Model

**Figure 1:**
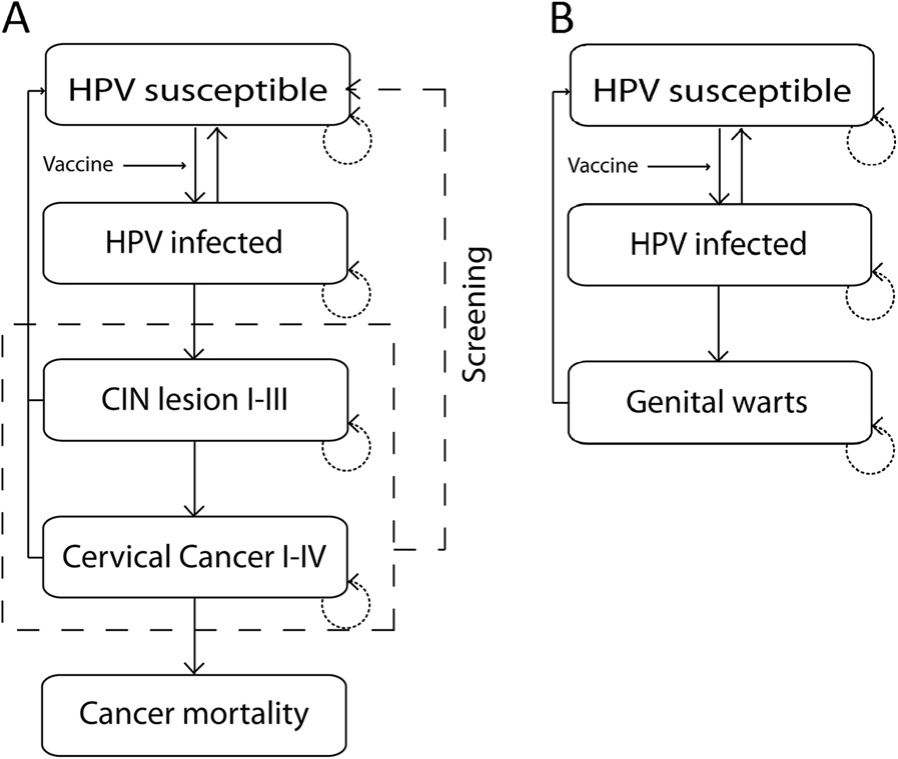
**Schematic representation of the progression-of-disease Markov models simulating the progression to cervical cancer (A) and genital warts (B). **Individuals progress or regress from one health state to another according to disease-specific transition probabilities (solid lines) or women can remain in the same health state during consecutive cycles (dotted lines). Vaccine efficacy was modelled by reducing the risk of infection. Cycle length was set at 6 months’.

A previously published Markov model for HPV infection and cervical cancer was modified to take the additional burden of genital warts into account [11]. The initial model structure (Figure 1A) and input data (Additional file 1) have been described in detail previously [11]. In the current modified model, the potential cross-protection benefits of both vaccines can be considered, since multiple HPV-types (e.g. HPV16, 18, 31, 33, 45, other high-risk HPV types and other low-risk HPV types) are included in the design. In addition, the modified model simulates the progression of HPV infection to genital warts for two categories of HPV types: “6/11” and “other low-risk types” (Figure 1B). The transition probabilities from “susceptible” to “HPV-infected” to “genital warts” were adapted from literature (Table 1 and Additional file 1) [14]. The modified model was calibrated to age- and type-specific HPV-prevalence and genital warts incidence (Table 2) [7,15-17]. The model was used to estimate the clinical and economical benefits of vaccination with either the bivalent or the quadrivalent HPV vaccine in the context of the NIP, in addition to the current Dutch cervical cancer screening program. Note that it was assumed that neither the sensitivity of the screening program nor the attendance is changed by the implementation of HPV vaccination.

**Table 1:** Epidemiologic and economic parameters used in the model

6 month transition probabilities*	**Mean and/or range**		**Reference**
Normal to HPV 6/11^¥^	0 – 0.007		model calibration
Normal to lrHPV^¥^	0 – 0.04		model calibration
HPV 6/11 to warts	0.34		[14]
lrHPV to warts	0.009		model calibration
Natural clearance HPV infection	0.65		[14]
Natural clearance genital warts	0.65		[16]
**Screening costs**	**Mean (€)**		
Cytology	50		[11]
Colposcopy	143		[11]
Biopsy	49		[11]
**Treatment costs cancer**	**Mean (€)**		
CIN 1	1,483		[11]
CIN 2	1,718		[11]
CIN 3	1,868		[11]
Cervical cancer stage 1	19,114		[11]
Cervical cancer stage 2	20,762		[11]
Cervical cancer stage 3	20,762		[11]
Cervical cancer stage 4	26,528		[11]
**Treatment costs warts**	**Mean (€)**		
GP	114		Table 3
STI clinic	285		Table 3
GP + STI clini	338		Table 3
**QALY-losses**	**Mean**		
CIN 1	0.026		[11]
CIN 2	0.010		[11]
CIN 3	0.080		[11]
Cervical cancer stage 1	0.03		[11]
Cervical cancer stage 2	0.10		[11]
Cervical cancer stage 3	0.10		[11]
Cervical cancer stage 4	0.38		[11]
Genital warts	0.018		[20]
**Vaccine characteristics**	**Bivalent**	**Quadrivalent**	
Efficacy HPV 16/18	95%	95%	[23]
Efficacy HPV 6/11	0%	95%	[23]
Cross-protection HPV 31	79%	57%	[4,5]
Cross-protection HPV 33	46%	0%	[4,5]
Cross-protection HPV 45	76%	0%	[4,5]
Duration of protection	lifelong	lifelong	[25]
Vaccination costs per dose	€105	€105	

¥ transition probabilities are age dependent.

* 6-months’ probability of moving from one health state to another.

NA = not applicable; GP = general practitioner; STI = sexually transmitted infection; CIN = cervical intraepithelial neoplasia; HPV = human papillomavirus.

**Table 2:** **Average annual age-specific number of reported genital warts cases (2002-2007) in women by GP or STI clinic **[7]

Age group	GP	STI clinic	Total
10 – 14	432	1	433
15 – 19	1,120	139	1,259
20 – 24	1,911	437	2,348
25 – 29	2,267	210	2,070
30 – 34	1,978	92	1,392
35 – 39	1,343	48	787
40 – 44	753	34	385
45 – 49	368	17	181
50 – 54	167	14	79
55+	196	4	200
Total	10,536	996	11,533

GP = general practitioner, STI = sexually transmitted infections.

### Burden of disease

Table 2 gives an overview of the age-specific numbers of reported cases of genital warts in women treated by GPs and STI-clinics, annually. In the Netherlands, the incidence of genital warts in the total female and male population is on average approximately 11,500 and 8,100 annually, respectively [7]. Of these cases approximately 9% are treated in clinics for sexually-transmitted infections.

No direct coherent Dutch data are available about the treatment costs of genital warts and the quality of life during an episode of genital warts. Therefore, costs were estimated from different sources, including national databases, literature and expert opinions [7]. The medical costs include general practitioner (GP) visits, specialist visits, treatment costs and pharmacists’ fees. The utility losses of genital warts were adapted from literature [18].

Total GP treatment costs of genital warts were estimated from the Netherlands Information Network of General Practice (LINH). Data available through LINH are based on electronic medical records from about 85 general practices, spread throughout the country. Data include longitudinal information on patient characteristics such as age, sex, degree of urbanization, as well as medical information on consultations, prescriptions, referrals, and diagnoses. GPs participating in the LINH network are instructed to use ICPC-1 codes for every patient contact. Based on ICPC-codes X91 and Y76, probable cases of genital warts in 2007, 2008 and 2009 were identified. A total of 935 (491 female and 444 male) patients with genital warts were identified over the period 2007-2009. Medication was prescribed in 80% and 91% for the female and male patients, respectively. Per treated patient an average of 1.7 prescriptions were counted (range 1 to 22). Podophylotoxin and imiquimod were the drugs mostly used, in 90% and 5% of the cases, respectively. Women diagnosed with genital warts visited their GP slightly more often than men, an average of 1.8 (range 1 to 20) and 1.5 (range 1 to 18) visits per person, respectively. Furthermore, on average, 0.2 (range 0 to 4) telephone consultancies per person diagnosed with genital warts were made. Surgical removal (CTG Dutch hospital costing codes 13012, 13047, 13048) of the warts was performed with 37 patients, requiring an average of 1.2 surgical interventions per patient. Finally, 115 (12%) patients were sent to a medical specialist, most often a gynaecologist (i.e. 40% of female patients) or a dermatologist (i.e. 60% of female and 90% of male patients). Unit costs were adapted from Oostenbrink *et al*. and from the Dutch Health-Care Insurance Board [19]. Summing up all the costs, the average per-patient GP-treatment costs were estimated at €114 and €106 for females (Table 3) and males, respectively. The treatment costs of genital warts within an STI-clinic (€285 per case) were obtained from expert consultation.

**Table 3:** Detailed build-up of the average per female patient treatment costs of genital warts

	**Unit costs (€)**	**GP (€)**	**STI clinic (€)**	**GP + STI clinic (€)**
GP visits	28	50.40	0	50.40
Telephone consult	14	2.80	0	2.80
Specialist visits^¥^	59	0	224.20	224.20
Podophylotoxin	35	42.80	42.80	42.80
Imiquimod	100	6.80	6.80	6.80
Pharmacist fee	5.50	5.50	5.50	5.50
Prescription	5.50	5.50	5.50	5.50
Chirurgical treatment^¥¥^	10.00	0.45	0	0.45
Total direct costs		114	285	338

GP = general practitioner, STI = sexually transmitted infections.

¥ In the Netherlands a medical specialist receives a fixed price for the treatment of genital warts.

^¥¥ ^Adapted from Woodhall et al. [20].

It has been shown that the quality of life during an episode of genital warts decreases to 0.944 [20,21]. This corresponds to a QALY loss of approximately 0.018 per case of genital warts [18].

Estimates of the costs and QALYs, for the different (pre-)cancer stages, were adapted from our previous publication (Table 1) [11]. For incidence data, treatment costs and quality-of-life estimates for cervical cancer and pre-cancer stages, we refer to our previous publication [11].

### Vaccine characteristics

The bivalent as well as the quadrivalent HPV vaccine shows a high efficacy against infection with HPV16 and 18, the virus types included in both vaccines. In line with our previous studies, we conservatively assumed a 95% efficacy against HPV16 and HPV18 infection for both vaccines [9]. Furthermore, both vaccines induce cross-protection against other oncogenic HPV-types [4,5,22-24]. Since the bivalent vaccine induces a higher degree of cross-protection than the quadrivalent vaccine (Table 1), vaccine-specific cross-protection efficacies were explicitly taken into account. Finally, it was assumed, that both vaccines induce lifelong protection [25]. Total vaccination costs were set at €315 per vaccinated woman (including 3 doses and administration costs). Note that relevant lower vaccine prices are paid within the Dutch NIP, and probably also in other national immunization programmes, after tendering. Therefore, vaccination costs were reduced in sensitivity analysis. No utility losses due to side effects were considered.

### Cost-effectiveness analyses

Differences in costs and effects were estimated by following a cohort (100,000 women) during life twice, once as a partly (e.g. 50%) vaccinated (bivalent or quadrivalent) cohort and once as an unvaccinated cohort. For all situations it was assumed that 90% of all women attend the current Dutch cervical cancer screening program at least once during life according to the “pre-vaccine-era” 5-yearly compliance rate of 80%. No changes in the screening program structure or compliance rates were assumed for the vaccinated cohort. The model tracks the total number of cases (e.g. cervical cancer, cervical premaligancies, and genital warts), costs, life-years (LY) gained and changes in QALYs. By summing up all the costs, LY gained or QALYs, the net costs, LYs and QALYs gained/lost were determined for both options. Subsequently, the difference between the vaccinated and unvaccinated cohort was determined. The incremental cost-effectiveness ratio (ICER) was calculated by dividing the incremental costs by the incremental LY or QALYs gained. In the Netherlands, interventions with an ICER below €20,000 per QALY gained are generally considered as being cost-effective. Future costs and health effects were discounted at 4% and 1.5%, respectively, reflecting the Dutch guidelines for cost-effectiveness research [26]. Furthermore, to enhance the transferability of our base-case findings to other countries, we also performed sensitivity analyses at costs and health outcomes discount rates of both 3%. Finally, the vaccine prices of the vaccines were varied to achieve equal ICERs for both.

### Sensitivity analyses

The robustness of the outcomes were analyzed in univariate sensitivity analyses. As there is uncertainty about the duration of vaccine-induce protection, vaccine price, cross-protection, and the treatment costs of genital warts, these parameters values were varied in univariate sensitivity analyses. To provide insight in the impact of possible additional herd-immunity benefits on our findings a certain degree of herd-immunity was incorporated in our model. In particular, based on a previous study of Bogaards *et al*., we estimated a relative risk reduction among unvaccinated women for developing cervical cancer of 33%, aligning a 50% vaccine uptake [8]. This risk reduction was applied to both the unvaccinated male and female populations for both cervical cancer and genital warts. As the herd-immunity benefit is highly sensitive to the vaccination coverage, these benefits were estimated for different coverages; i.e. for 50%, 70% and 90% coverage, the relative risk reductions were estimated at 33%, 55% and 84%, respectively. Finally, to enhance the transferability of our finding to other countries the discount rate and screening compliance was varied in sensitivity analyses. As the Netherlands and Belgium are unique in discounting health effects with a lower rate compared to costs, the discount rates for both health effects and costs were also varied independently in the sensitivity analyses. Furthermore, in the Netherlands 90% of women are screened at least once during life. Compared to other countries this is a relative high coverage and therefore we decreased this coverage to 70%, 50% and 30% in sensitivity analysis.

In addition to the impact of the above parameters on the estimated ICER, we also determined the price difference between the two vaccines under conditions of equal ICERs, not only for the base case, but also for the different options analyzed in the sensitivity analyses.

## Results

### Clinical results

Introduction of HPV vaccination for 12-year-old girls in the model, at a 50% vaccination coverage reduces the annual HPV16/18-related cervical cancer incidence by 198 cases, independent of which vaccine is used. Due to the benefits of cross-protection [5], the bivalent vaccine will prevent 23 cases of cervical cancer induced by one of the other high-risk HPV types while, due to a lower level of cross-protection [6] thusfar documented, the quadrivalent vaccine will prevent 9 cases due to cross-protection. So, in total we estimated that the bivalent and quadrivalent HPV vaccine prevent 221 and 207 cases of cervical cancer, respectively. Furthermore, both vaccines will prevent cervical premalignancies, the bivalent vaccine 106 CIN1, 203 CIN2 and 264 CIN3, and the quadrivalent vaccine 91 CIN1, 182 CIN2 and 237 CIN3. In addition, the quadrivalent vaccine will prevent annually 4,390 cases of genital warts.

### Cost-effectiveness of HPV vaccination

Notably, 1,524 (646 if discounted) or 1,430 (606 if discounted) life years will be gained when the bivalent or quadrivalent HPV vaccine are being used, respectively. When all the above health benefits are taken together, in total 1,815 (790 if discounted) or 1,803 (824 if discounted) QALYs are estimated to be gained by using the bivalent or the quadrivalent vaccine, respectively. In particular, for the bivalent vaccine 709 discounted QALYs are gained by providing protection against HPV16/18-induced cervical (pre-)malignancies and 81 discounted QALYs are gained due to cross-protection. For the quadrivalent vaccine, 709, 33 and 82 discounted QALYs are gained due to protection against HPV16/18-induced (pre-)malignancies, cross-protection and prevention of genital warts, respectively. Furthermore, vaccination with the bivalent vaccine results in a discounted cost-saving of €1,641,000 by providing protection against HPV16/18-induced (pre-)malignancies and discounted cost-saving of €211,000 due to cross-protection. The quadrivalent vaccine results in discounted cost-savings of €1,641,000, €84,000 and €570,000 due to protection against HPV16/18-induced (pre-)malignancies, cross-protection, and prevention of genital warts, respectively. The total vaccination costs are estimated at €15M per year in the Netherlands.

Considering only the benefits of prevention of HPV16/18-related disease, the ICER was estimated at €19,900 per QALY or €24,300 per LY gained (€41,900/QALY or €52,800/LY if discounted at 3%). When the benefits of cross-protection and protection against genital warts are taken into account, the ICER of the bivalent and quadrivalent vaccines were estimated at €17,600 per QALY or €21,500 per LY gained (€36,900/QALY or €46,500/LY if discounted at 3%) and €16,300 per QALY or €22,700 per LY gained (€31,800/QALY or €49,600/LY if discounted at 3%), respectively.

### Sensitivity analyses

**Figure 2:**
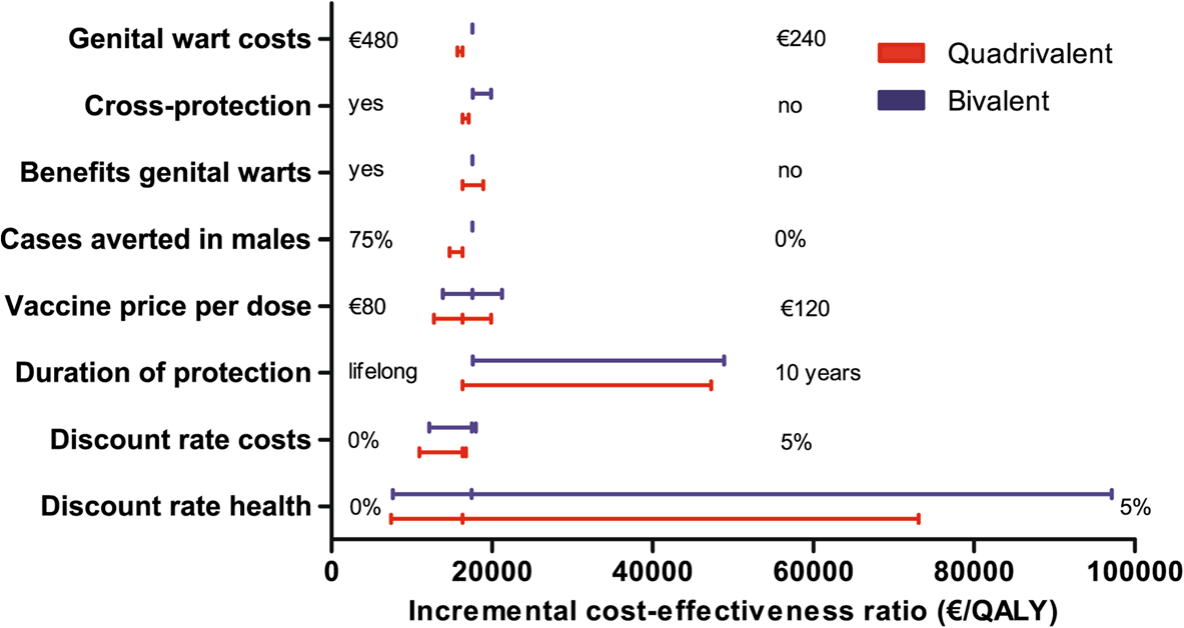
Incremental cost-effectiveness ratio of varying parameter values in univariate sensitivity analyses.

**Figure 3:**
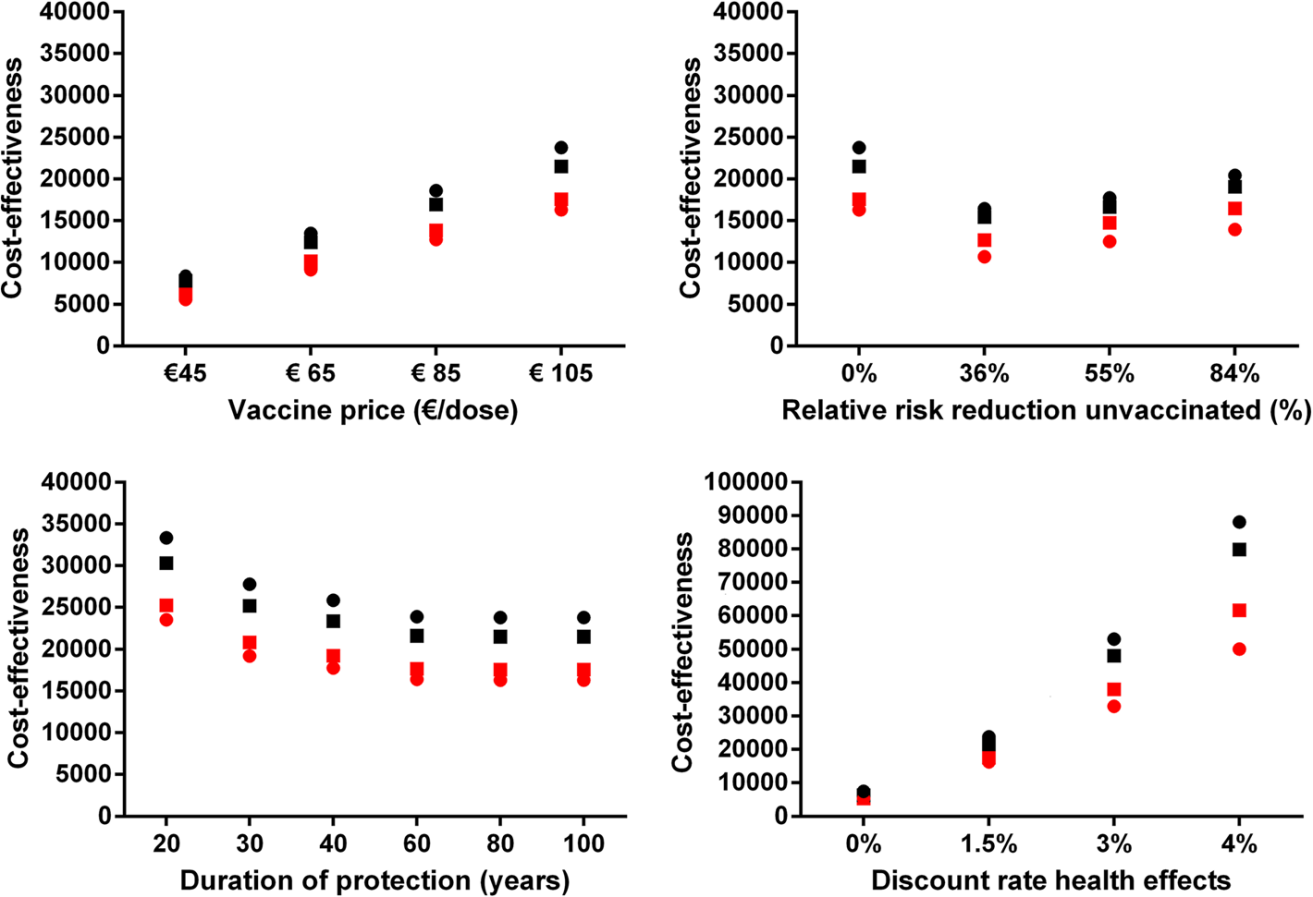
**Cost-effectiveness (Euro/QALY and Euro/LY) for the bivalent and quadrivalent HPV vaccine. **Black: Euros per life year gained; red: Euros per quality-adjusted life years gained; circles: quadrivalent vaccine; squares: bivalent vaccine.

**Figure 4:**
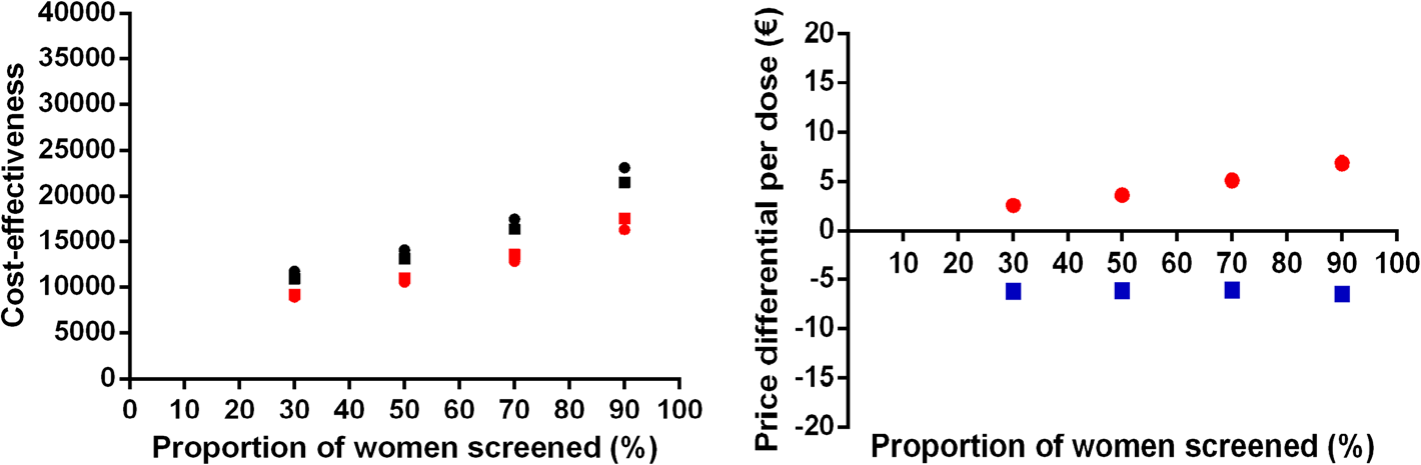
**Cost-effectiveness and price differential of HPV vaccines in setting with a reduced screening compliance. **Left panel shows cost-effectiveness of HPV vaccination. Black: € per life-year gained; red: € per quality-adjusted life years gained. Circles: quadrivalent vaccine; squares: bivalent vaccine. Right panel: price differential between bivalent and quadrivalent HPV vaccine. Blue squares: price differential considering the life-years gained; red dots: idem if quality-adjusted life years gained are considered.

In the univariate sensitivity analyses, the ICER for the bivalent HPV vaccine ranged from €2,200 to €97,100 per QALY gained. The ICER for the quadrivalent vaccine ranged from €1,000 to €73,100 per QALY gained. Figure 2 shows the upper and lower limit of the ICER upon variation of individual parameter values. Figure 2 shows that the ICER was most sensitive to vaccine price, duration of protection, herd-immunity and the discount rate for health benefits. Figure 3 provides a detailed overview of the cost-effectiveness of HPV vaccination for the four abovementioned scenarios. Also, we considered a best-case (i.e. vaccine price €45/dose, lifelong protection, herd immunity and discount rates costs and health effects at 0%) and a worst-case scenario (i.e. vaccine price €105, 20-years protection, no herd immunity, and high discount rates cost and health effects at 4%). HPV vaccination was estimated to be cost-saving (irrespective of the HPV vaccine) in the best-case scenario. In the worst-case scenario the ICER was €81,800/QALY (€107,700/LY) or €66,500/QALY (108,200/LY), for the bivalent and quadrivalent HPV vaccine, respectively. Finally, the cost effectiveness of HPV vaccination was found to be more favourable in a setting with a moderate or poor screening program (Figure 4).

### Price differential between the bivalent and quadrivalent HPV vaccine

**Figure 5:**
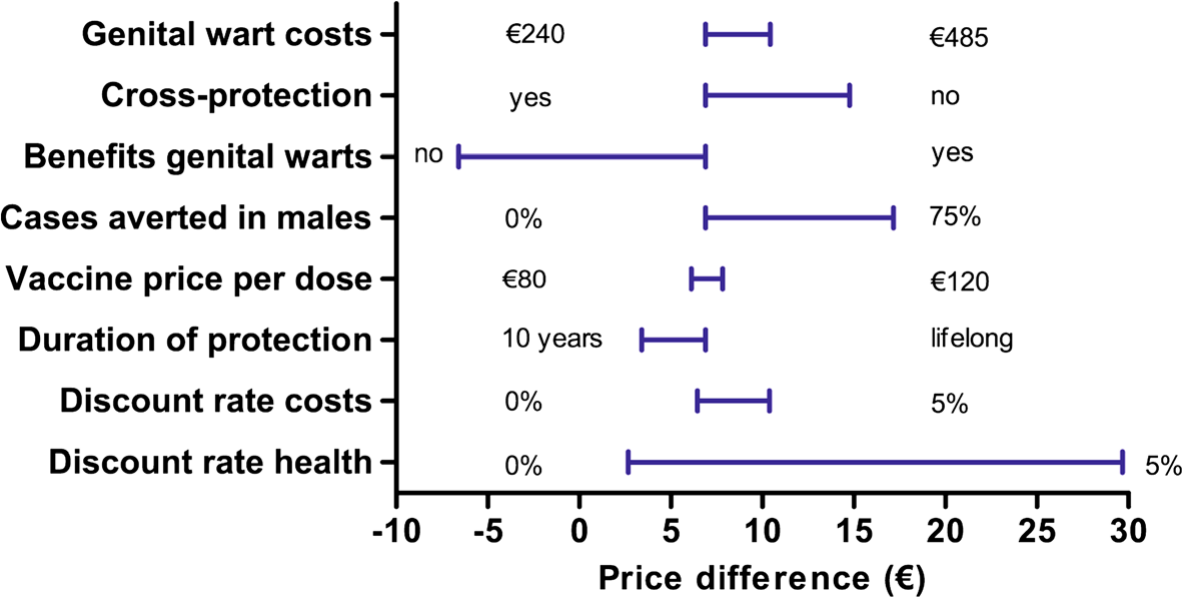
**Sensitivity analyses on the price differential of the bivalent and quadrivalent HPV-vaccines. **A positive price difference indicates that the quadrivalent vaccine can be more expensive to be as cost-effective as the bivalent vaccine.

**Figure 6:**
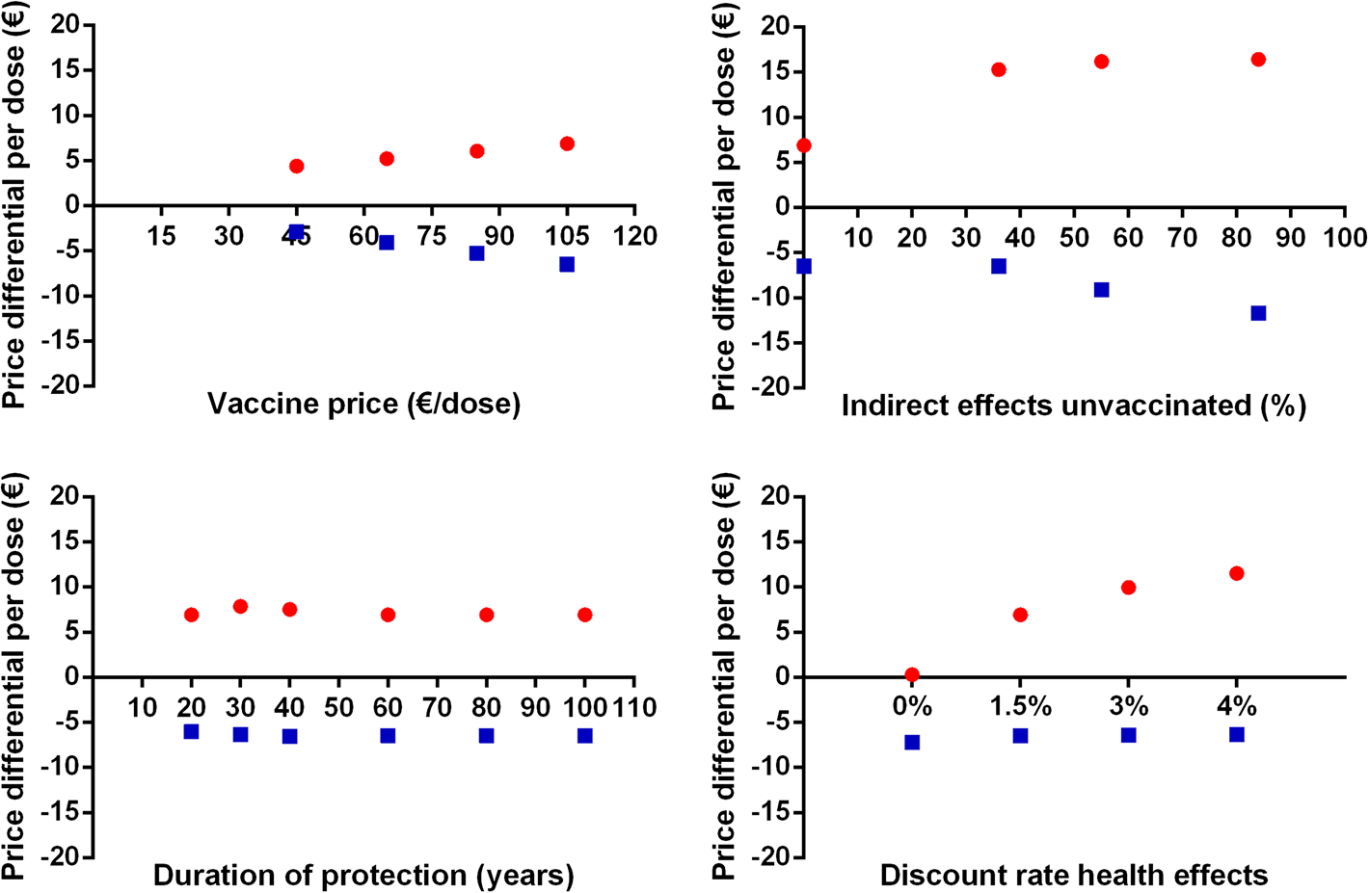
**Price differential for the quadrivalent vaccine to be as cost-effective as the bivalent vaccine. **Blue squares indicate the price differential considering the life-years gained and red dots indicate the same if quality-adjusted life years gained are considered.

The per-dose price differential between both vaccines was estimated such that the cost-effectiveness of the bivalent vaccine would equal that of the quadrivalent vaccine (€16,300 per QALY gained). Note that, here, we considered an equal willingness-to-pay for prevention of cancer and genital warts. In the base-case, we estimated that, based on a list price of €105/dose, the bivalent vaccine has to be €6.90 per dose less expensive compared to the quadrivalent vaccine to achieve an equal ICER (€14, if discounted at 3%). This price difference was most sensitive to assumptions about herd immunity, treatment costs of genital warts, exclusion of the benefits of either cross-protection or genital warts, vaccination costs and, again, the discount rate for health benefits (Figures 5 and 6). The price differential ranged from minus €2,80, if only life years gained were considered (i.e. the bivalent vaccine might be more expensive), to almost €11 if future health outcomes are discounted at 4%. Indeed, consideration of herd immunity resulted in a larger price difference between both vaccines to achieve equal ICERs. A 33% reduction in incidence of genital warts and cervical cancer among unvaccinated females and males resulted in a price differential of approximately €15 per dose. If vaccine coverage would further increase to 90%, the price differential increased to €16 per dose. Finally, if the bivalent vaccine provides an extended duration of protection compared to the quadrivalent vaccine, as has been hypothesized by Einstein *et al*. [27], the results were in favour of the bivalent vaccine. In particular, assuming lifelong protection for the bivalent vaccine and 20 or 40 years for the quadrivalent vaccine, we estimated a price differential of €24 and €1 per dose, respectively. Finally, in the worst-case scenario, the price differential was estimated at €21.

In settings with a moderate or poor screening program the estimated ICER was more driven by the health gains of providing protection against cervical cancer. Consequently, the price differential diminishes in settings with a higher burden of cervical cancer, often accompanying a relatively poor screening program (Figure 4).

There are three scenarios in which the bivalent vaccine may have a higher price compared to the quadrivalent vaccine to be equally cost-effective. These scenarios include the conditions in which (i) only LY gained are considered, (ii) the quadrivalent vaccine provides a shorter duration of protection, and (iii) prevention of genital warts are not taken into account.

## Discussion

Currently, there are two registered prophylactic HPV vaccines, a bivalent vaccine and a quadrivalent vaccine. To decide which vaccine could best be used in countries’ national immunization programs, an analytical comparison in terms of cost-effectiveness between both vaccines is required. Accordingly, the primary aim of the current study was to make a specific comparison between the bivalent and the quadrivalent HPV vaccines, taking the specific characteristics of both vaccines explicitly into account. These include not only the benefits of protection against HPV16 and 18, but also those of cross-protection against other high-risk HPV types and, for the low-risk HPV types, protection against genital warts. Note that, although differences in immunogenicity between both vaccines have been reported [24], the clinical relevance of this has not been demonstrated yet. Even though we conducted the study for the situation in the Netherlands, the general conclusions of our analyses are likely to apply, with minor modifications, to other European countries and the USA, because of strong similarities in HPV prevalence and implementation of prophylactic vaccination programs in these countries.

From our base-case analyses, it follows that use of the bivalent HPV vaccine gives the highest reduction in cervical cancer incidence. Consequently, the highest number of life-years gained was obtained by implementing the bivalent vaccine, resulting in a more favourable ICER compared to that obtained with the quadriavalent vaccine (€21,500/LYG vs €22,700/LYG). This implies that the bivalent vaccine might be €2.80 more expensive if only LYs are considered. Considering QALYs gained on the basis of protection against (pre-)cervical malignancies and genital warts, the quadrivalent vaccine provided the highest health gain. The ICER of the quadrivalent vaccine was estimated to be more favourable than that of the bivalent vaccine at €16,300/QALY gained vs. €17,600/QALY. This implies that the quadrivalent vaccine can be approximately €7 per dose (based at a list price of €105/dose) more expensive than the bivalent vaccine to be equally cost-effective.

The ICERs of both vaccines were found to be particularly sensitive to the vaccine price (range: €5,600 - 17,600/QALY), the duration of protection (range: €16,300 - 48,900/QALY), the discount rate for health benefits (range: €5,300 - 97,100/QALY) and, to herd-immunity benefits (range: €10,700 - 17,600/QALY). In most scenarios, the ICER (€/QALY) of the quadrivalent vaccine remained more favourable than that of the bivalent vaccine. This difference, as argued above, may justify a slightly higher vaccine price for the quadrivalent vaccine. Interestingly, without discounting, the bivalent vaccine results in a slightly higher QALY gain than the quadrivalent vaccine, despite the benefits in terms of prevention of genital warts of the latter. This illustrates that the health gains of prevention of cervical cancer prevention are highly sensitive to the discount rate applied. Furthermore, it is important to note that the acceptable price differential between both vaccines highly depends on the vaccine price applied for the reference vaccine. In particular, in the base-case (bivalent vaccination costs was €105/dose), the price differential was estimated at €7 per dose. Reducing the vaccination costs of the bivalent vaccine to, for example, €45 per dose, the price differential decreases to €4. Possible lower vaccine costs will further reduce the price differential between both vaccines. Inclusion of additional herd-immunity benefits resulted in an increased price difference between both vaccines primarily due to the benefits of providing indirect protection against genital warts in males. Interestingly, the absolute herd-immunity benefit in females will be highest if vaccine coverage is moderate, while in males the herd-immunity benefit will highest when vaccine uptake is high.

In our study, the indirect effects could not be directly considered as a static model was used. The use of static models for infectious disease modelling has been criticised by us and others [28-30]. However, in balancing complexity and transparency, static models are still of major importance and can be used for initial assessments. Here, we included the potential herd-immunity benefits based on a previous Dutch modelling study [23]. Further research should be directed to develop a dynamic transmission model including the most important high- and low-risk HPV types. Another limitation of the current study is that potential additional health benefits of providing protection against other than cervical cancers were not included. The inclusion of these cancers will further improve the ICER for both vaccines. Consequently, price differentials between both vaccines might diminish [18], as the benefit of providing protection against genital warts will play a less prominent role. Finally, we did not perform any probabilistic sensitivity analysis (PSA). The use of PSA is highly recommended as it provides some insight in the certainty of the outcomes. However, comparing the PSAs of different vaccines is rather critical as potentially a highly favourable option (upper limit 95% confidence interval) of one vaccine is compared with a rather unfavourable option (lower limit 95% confidence interval) of the other vaccine. For that reason we currently didn’t embark on a formal PSA, but rather performed an extensive deterministic sensitivity analysis.

Our results are generally in line with the findings of others. In particular, several studies estimated that the bivalent HPV vaccine resulted in the highest reduction in cervical cancer incidence due to the additional benefits of cross-protection [28,30]. However, as only the quadrivalent vaccine provides protection against genital warts, the decision which vaccine to use is not that straightforward. From a health-economic perspective, the most cost-effective vaccine should be preferred considering both the health gains of providing protection against cervical cancer and genital warts. Several studies made this type of comparison between both vaccines [25-28]. Here, we estimated that a €6.70 (7%) price differential, in favour of the quadrivalent HPV vaccine, resulted in equal ICERs. This justifiable price difference between both vaccines estimated in the present study is slightly different from that found in other studies [31]. For example, Jit *et al*. estimated that the bivalent vaccine should be approximately 25% less expensive [32]. Indeed, even larger discrepancies have been reported [31,33]. The difference between our study and those of others can be explained by various factors. Firstly, as mentioned above, we used a static model in which herd-immunity benefits are not directly taken into account. As indicate above, inclusion of herd-immunity benefits results in an increased price difference (e.g., the price difference reaches a maximum of approximately 16% when herd-immunity is taken into account). Secondly, we included vaccine-specific cross-protection against high-risk HPVs in our analyses. As there is evidence that the bivalent vaccine is more cross-protective than the quadrivalent vaccine [5,6], this results in a smaller price difference. Finally, according to health-economic guidelines in the Netherlands, future health benefits are discounted with a relatively low discount rate of 1.5%, and therefore, the health benefits of cervical cancer are devalued less than in most other countries. Nevertheless, in general we can conclude that in western countries a higher vaccine price for the quadrivalent vaccine is justified due to the additional benefits of providing protection against genital warts.

In contrast, Demarteau *et al*. estimated that for Taiwan a higher vaccine price for the bivalent vaccine is justified [34]. The difference between this study and the studies performed in western countries can be primarily explained by the higher incidence of cervical cancer in Taiwan. Due to the potentially higher effectiveness of the bivalent vaccine in providing protection against cervical cancer a relevantly higher health gain might be obtained in settings with a high burden of cervical cancer. In most western countries the burden of cervical cancer has already been dramatically reduced since the introduction of cervical cancer screening. Consequently, in these settings the benefits of providing protection against genital warts are predominant and the ICER of the quadrivalent vaccine is even more sensitive to the inclusion of the benefits of providing protection against genital warts than found in our study. This illustrates that the decision which vaccine to use on health-economic grounds will highly depend on the disease burden of cervical cancer versus the burden of genital warts.

## Conclusion

In conclusion, the results of our study demonstrate that HPV vaccination is a cost-effective intervention in the Netherlands. Clearly, HPV vaccination has been implemented for the prevention of cervical cancer. From this perspective, use of the bivalent HPV vaccine appears to be most effective and most cost-effective in prevention of cervical cancer. This is primarily due to the broader cross-protective capacity of the bivalent vaccine, compared to the quadrivalent vaccine, against high-risk HPV types not included in the vaccines. However, if the potential benefits of providing protection against genital warts are considered, another decision might be made. From a health economic perspective, a coherent analytic comparison of the bivalent and quadrivalent HPV vaccines could be made and to consider costs and benefits of cross-protection against other high-risk HPV types not included in the vaccines as well as those of protection against genital warts. From a health care decision-making perspective, these analyses – next to other criteria – can then be compared to the initial intention of cancer prevention underlying introduction of the vaccination in local national vaccination programs. This will then provide a more balanced view than cost-comparison alone.

## Competing interest

None of the authors have any competing interest. MJP and TAW received research and travel grants from GlaxoSmithKline. MJP received grants from Sanofi MSD. TAW is currently working for GlaxoSmithKline. This work is from the period TAW worked for the University Medical Center Groningen.

## Authors’ contribution

MJP, TAW, HWN, TD and JCW study design, TAW, ELdV, EDT, IS, SD data collection, TAW, IS and SD data analysis, TAW, ELdV and EDT model design and calculations, TAW, ELdV, EDT and IS writing manuscript, MJP, JCW, HWN and TD critically reviewing manuscript. All authors read and approved the final manuscript.

## Pre-publication history

The pre-publication history for this paper can be accessed here:

http://www.biomedcentral.com/1471-2334/13/75/prepub

## Supplementary Material

Additional file 1 HPV-type specific cervical cancer progression and regression 6 month progression rates. Click here for file
