# Supplementary material for: Inclusion of the benefits of enhanced cross-protection against cervical cancer and prevention of genital warts in the cost-effectiveness analysis of human papillomavirus vaccination in the Netherlands
Source: BMC Infect Dis. 2013 Feb 7;13:75. doi: 10.1186/1471-2334-13-75 (PMC3575363; doi:10.1186/1471-2334-13-75)
Supplement: Additional file 1 — HPV-type specific cervical cancer progression and regression 6 month progression rates. [file 1471-2334-13-75-S1.docx]

|  | HPV16 | HPV18 | HPV31 | HPV33 | HPV45 | Other Hr | Other Lr |
| --- | --- | --- | --- | --- | --- | --- | --- |
| Risk of infection | 0.0019 - 0.01425 | 0.0007 – 0.0075 | 0.0007 – 0.0023 | 0.0001 – 0.0008 | 0.0001 – 0.0023 | 0.0021 – 0.0210 | 0.0024 – 0.0240 |
| Infected to normal | 0.38 | 0.38 | 0.38 | 0.38 | 0.38 | 0.38 | 0.53 |
| Infected to CIN1 | 0.04 – 0.05 | 0.032 – 0.04 | 0.032 – 0.04 | 0.032 – 0.04 | 0.032 – 0.04 | 0.032 – 0.04 | 0.016 – 0.02 |
| Infected to CIN2 | 0.004 | 0.004 | 0.0034 | 0.0034 | 0.0034 | 0.0034 | 0.0009 |
| CIN1 to CIN2 | 0.09 – 0.15 | 0.045 – 0.075 | 0.0113 – 0.0188 | 0.0113 – 0.0188 | 0.0113 – 0.0188 | 0.0113 – 0.0188 | 0.0056 – 0.0094 |
| CIN1 to CIN3 | 0.032 – 0.0533 | 0.016 – 0.0266 | 0.004 – 0.0067 | 0.004 – 0.0067 | 0.004 – 0.0067 | 0.004 – 0.0067 | 0 |
| CIN1 to normal | 0.3 – 0.5 | 0.3 - 0.5 | 0.3 – 0.5 | 0.3 – 0.5 | 0.3 – 0.5 | 0.3 – 0.5 | 0.3 – 0.5 |
| CIN2 to normal | 0.02 | 0.02 | 0.02 | 0.02 | 0.02 | 0.02 | 0.02 |
| CIN2 to CIN3 | 0.03 – 0.10 | 0.03 – 0.10 | 0.03 – 0.10 | 0.03 – 0.10 | 0.03 – 0.10 | 0.03 – 0.10 | 0.03 – 0.10 |
| CIN3 to cancer stage 1 | 0.006 – 0.0690 | 0.0043 – 0.0587 | 0.0026 – 0.0255 | 0.0026 – 0.0255 | 0.0026 – 0.0255 | 0.0026 – 0.0255 | 0.0008 – 0.0106 |
| CIN3 to normal | 0.02 | 0.02 | 0.02 | 0.02 | 0.02 | 0.02 | 0.02 |
| CIN3 to CIN2 | 0.005 | 0.005 | 0.005 | 0.005 | 0.005 | 0.005 | 0.005 |
| Stage 1 to stage 2 | 0.11 | 0.11 | 0.11 | 0.11 | 0.11 | 0.11 | 0.11 |
| Stage 2 to stage 3 | 0.12 | 0.12 | 0.12 | 0.12 | 0.12 | 0.12 | 0.12 |
| Stage 3 to stage 4 | 0.12 | 0.12 | 0.12 | 0.12 | 0.12 | 0.12 | 0.12 |
| Mortality stage 1 | 0.002 – 0.007 | 0.002 – 0.007 | 0.002 – 0.007 | 0.002 – 0.007 | 0.002 – 0.007 | 0.002 – 0.007 | 0.002 – 0.007 |
| Mortality stage 2 | 0.01 – 0.02 | 0.01 – 0.02 | 0.01 – 0.02 | 0.01 – 0.02 | 0.01 – 0.02 | 0.01 – 0.02 | 0.01 – 0.02 |
| Mortality stage 3 | 0.03 – 0.042 | 0.03 – 0.042 | 0.03 – 0.042 | 0.03 – 0.042 | 0.03 – 0.042 | 0.03 – 0.042 | 0.03 – 0.042 |
| Mortality stage 4 | 0.06 – 0.07 | 0.06 – 0.07 | 0.06 – 0.07 | 0.06 – 0.07 | 0.06 – 0.07 | 0.06 – 0.07 | 0.06 – 0.07 |

Appendix: HPV-type specific cervical cancer progression and regression 6 month progression rates
